# “A creature inside me”: perceptions and representations of HIV among adolescents living with HIV in Malawi

**DOI:** 10.1186/s12981-025-00770-4

**Published:** 2025-07-26

**Authors:** Nadine Ammon, Mark Limmer, Alex Kaley

**Affiliations:** 1 Lancaster University, Bailrigg, Lancaster, LA1 4YW UK; 2 Health Research Department, Lancaster University, Lancaster, UK; 3 University of East Anglia, Norwich, UK

**Keywords:** Adolescents, HIV, Education, Language, Perceptions, Drawings

## Abstract

Malawi is among the countries with the highest HIV prevalence worldwide. Adolescents living with HIV (ALHIV) face diverse challenges, which influence their emotional wellbeing and long-term health, in addition to impacting HIV onward transmission. HIV education, especially the use of fear-based animation, but also the figurative language used for HIV, contribute to how ALHIV perceive and respond to their HIV status. The aim of the study was to explore how ALHIV in Malawi describe, perceive, and represent HIV, with a particular focus on the role of language in shaping these perceptions and its impact on their experiences and emotional wellbeing. This study employed hermeneutic phenomenology and reflexive thematic analysis; data were collected through semi-structured in-depth interviews, focus group discussions and drawings. Participants were sampled purposively and included 16 ALHIV and five service providers. The adolescents imagined HIV as personified, harmful creature in their body with functional senses and gender identity. Those negative perceptions originated mainly from the local term used for HIV, HIV-related stigma and discrimination and HIV representations in hospital HIV books. HIV peer support groups were identified as safe environments for learning about HIV and for debating HIV-related topics, especially in view of the usually required silence and secrecy to prevent stigma. The findings enhance the understanding of participants’ lived experiences and perceptions of HIV, and thus may contribute to new methods of holistic health education, tailored for adolescents to improve their emotional wellbeing and attitudes towards HIV through context-specific programmes.

## Introduction

The number of adolescents living with HIV (ALHIV), is estimated to be 1.74 million worldwide, with 60% living in Eastern and Southern Africa [1]. Furthermore, the HIV-related mortality rate among ALHIV has increased, reaching a crescendo as the leading cause of death among this group in SSA [2]. Malawi falls under the ten countries with the highest HIV prevalence among adolescents [3] with the main mode of transmission being either through mother-to-child transmission or sexual intercourse [4]. Considering that 50% of Malawi’s population are aged below 18 years [5] and only one in five adolescents gets tested for HIV [6], undiagnosed HIV further increases the probability of HIV onward transmission [7]. Since antiretroviral therapy (ART) has transformed the prognosis of people living with HIV (PLHIV) by achieving and maintaining an undetectable viral load through ART adherence, the risk of onward HIV transmission can be eliminated [8]. As such, ART plays a central role in the control of the HIV epidemic (Treatment as Prevention) [9]. Nevertheless, not all individuals who need ART are yet receiving treatment, therefore further efforts are required to scale up ART access for children and ALHIV [10].

Adolescence, defined as the developmental phase of 10–19 years [11], can further be divided into early adolescence (10–14 years) and late adolescence (15–19 years) [12]. Recognising their physical, emotional, mental and social changes, this is a demanding period, progressing from being a child to an independent adult. For ALHIV this is especially true, as in addition to the developmental changes, they are required to accept their chronic and extremely stigmatised condition. This explains the high rate of failure for adherence to lifelong ART and retention in HIV care [13].

For many ALHIV in SSA, who are often vulnerable due to disadvantaged life circumstances, adhering to ART remains a significant challenge [14, 15]. Factors contributing to inadequate ART adherence are multiple and complex. A systematic review identified barriers to optimal ART adherence, including forgetfulness, lack of support structures, insufficient age-appropriate health services, and also cultural and religious beliefs [16]. Additional challenges faced by ALHIV include economic hardship [17], orphanhood, issues related to disclosure, mental health conditions such as depression [18, 19], and experiences of stigma and discrimination [18, 20]. Regarding disclosure, the WHO [21] recommends a gradual introduction to HIV-related information rather than a one-time event, allowing for ongoing discussions until the adolescent demonstrates sufficient maturity and emotional stability to cope with the diagnosis.

### The role of Language and education in shaping HIV perceptions and wellbeing among ALHIV

HIV-related stigma fuels discrimination against PLHIV [22] with the distinction between enacted stigma experienced from others, anticipated or expected stigma, and internalised stigma, referring to self-discrimination [23]. The platforms where ALHIV experience HIV-related stigma include the family [24], schools [25], communities [26], and also health facilities [27]. Due to fear of stigma and rejection, ALHIV often avoid disclosing their HIV status to friends, intimate partners, and even family members, placing themselves and their intimate partners at risk of HIV transmission, emotional distress, and a lack of necessary support [28]. Furthermore, HIV-related stigma was found to be associated with poor mental health outcomes among ALHIV [29]. Particularly in regard to mental health, ALHIV’s wellbeing as a combination of mental and physical health, needs frequent follow-ups in order to offer mental health services timely. HIV-related stigma contributes to health inequalities [30], is a global concern and originates from lack of education, fear and outdated misconceptions traced back to the early years of the HIV epidemic. While mental health services in Malawi are limited [31], the prevalence of depression among ALHIV has been found to be 18.9% [32]. Hence, understanding and support from caregivers, the community and the health system is essential [33].

In contrast to the positive and encouraging information ALHIV receive at the HIV peer support group, more fear-based lessons about HIV are disseminated in schools, creating a knowledge barrier between ALHIV and their HIV-negative peers [34]. Recognising that school curricula are developed with the aim to prevent HIV, the need for pupils living with or affected by HIV in the classrooms are frequently neglected. For those, receiving contradictory messages about HIV, such as “living positively with HIV” at the hospital and “HIV as a killer” at schools, causes not only confusion, but also creates a division between HIV-positive and HIV-negative populations. In light of HIV now being a manageable condition, HIV-related communication, including formal education in schools, would benefit from adopting a broader and more critical perspective that carefully examines the potential impact of such messaging on HIV-related stigma [35]. Also, visual aids used for HIV education in schools and health facilities may influence the perception of ALHIV. The use of animated films and booklets for adolescents in SSA has shown to improve knowledge regarding HIV [36]. While visual aids were found beneficial for explaining complex concepts, most animations make use of scary metaphors, such as monsters, to illustrate and explain HIV [37], which may create misconceptions [38]. Misinterpretations of visual education material has been demonstrated by a study involving South African adults, in which pictorial metaphors, such as the “HIV monster” were misunderstood [39]. Furthermore, cultural and religious norms, myths and misconceptions may shape the experiences, perceptions and realities of ALHIV. Beliefs, such as “HIV as punishment from God”, “HIV is soiling people inside”, “HIV is spread through witchcraft” or “HIV can be cured by prayers” nourish HIV-related stigmatisation and compromise the wellbeing and treatment adherence of ALHIV [40, 41]. Consequently, it is important to note that adolescent wellbeing, in line with the Sustainable Development Goal 3 “To ensure healthy lives and promote wellbeing for all at all ages” [42], positively impacts health outcomes in adulthood [43].

An art therapy workshop facilitated in 2016 in Southern Malawi to understand ART adherence and disclosure behaviour in ALHIV, found that the majority of participants had negative perceptions about the image of HIV [44]. The ALHIV drew monsters, snakes and ant-like creatures when asked to illustrate the virus, which might be associated with the “small beast”, the Malawian term used for HIV. However, showing an electron microscopic image of HIV during discussion brought very positive feedback and was found much less frightening. Similarly, investigations into children’s views of cancer illuminated imagined monsters [45], which was also the case with adults, whose drawings of their tumours depicted monsters [46] and HIV has been portrayed as “gremlin” [47], illustrating illness perceptions.

Furthermore, the language used for certain conditions has an impact on illness perceptions, and as such, influences health behaviour [48, 49]. Therefore, health beliefs, illness perceptions and terminologies used for certain conditions evidently impact health-seeking behaviour and treatment adherence, as well as mental and physical health outcomes [50]. Using fear appeals in health communication to stir behaviour change has hitherto provoked controversial debates regarding its ethical appropriateness and usefulness [51], thus, numerous researchers recommended examining fear appeals in relation to their adverse psychological and ethical consequences [52, 53]. With the growing number of ALHIV in SSA and therefore an increased prerequisite for age-appropriate interventions, it is disputable whether the use of fear appeals in HIV communication facilitates HIV acceptance and emotional wellbeing, and whether or not it is beneficial in effecting the desired treatment adherence.

Most research conducted with ALHIV focused on HIV incidence, prevalence and behaviour [21, 54]. However, evidence regarding HIV education and ALHIV’s perceptions is more limited [55]. Furthermore, very few studies have investigated the lived experiences and perceptions of ALHIV in SSA linked to their imagination of HIV and their emotional wellbeing. Hence, a study, which gives ALHIV a voice and allows a creative form of expression through arts-based research methods could enhance a deeper understanding. Therefore, this study intended to inform health education interventions which are more appropriately tailored for adolescents to improve their emotional wellbeing and attitudes towards HIV through context-specific programmes, which in turn may have an impact on the control of HIV.

### Aim

The aim of the research was to explore descriptions, perceptions and representations of HIV among ALHIV, and to examine the impact of these interpretations on their emotional wellbeing.

### Objectives

The identified objectives to accomplish the aim of this research were to:

1. explore the language used for HIV, and the lived experiences and perceptions of ALHIV regarding HIV through conducting in-depth interviews;
2. investigate ALHIV’s imagination of HIV through drawings and the impact of the visual products on their emotional wellbeing;
3. facilitate focus group discussions among ALHIV to discuss their visual products and their underlying meanings ascribed to these images;
4. elucidate on and contextualise the interpretations of service providers on ALHIV’s visual products through a focus group discussion;
5. inform health education interventions that are tailored for adolescents to improve their emotional wellbeing and attitudes towards HIV through context-specific programmes.

### Research question

The research question guiding the study was:

In what ways do adolescents living with HIV in Malawi imagine HIV and how do these images and the language used for HIV impact on their emotional wellbeing, perceptions and lived experiences?

### Methods

In consideration of the study’s focus on subjective knowledge, an inductive, qualitative phenomenological methodology was used to explore ALHIV’s lived experiences and perceptions, and the interplays with their emotional wellbeing. Therefore, this study was positioned within a constructivist paradigm with a relativist ontology and subjectivist epistemology to appreciate the value-laden, pluralistic realities and their shared meanings, which depend on the context and are constructed and expressed through social exchanges and the use of language [56]. However, the researcher’s sympathetic and close contact and interaction with the participants during data collection influenced their realities throughout the research, and thus, the researcher’s reflexivity regarding the own contributions to the findings was vital [57].

### Hermeneutic phenomenology

Interpretive hermeneutic phenomenology, as proposed by Heidegger [58], aims to interpret and understand complex phenomena within the context they are occurring [59] and with a focus on language [60]. Since this research not only sought to present a description of ALHIV’s lived experiences and perceptions, but to explore and interpret these experiences and ALHIV’s reflection on these experiences in consideration of the underlying meaning, this approach was found suitable [61].

### Research setting

The study was conducted in Malawi`s capital city Lilongwe in one of the main urban HIV clinics, which has extensive experience in caring for children and ALHIV. The availability of a peer support group for ALHIV made this setting convenient for recruitment and data collection.

### Research population, eligibility criteria and sample size

Research participants consisted of ALHIV and service providers working at this clinic. The eligibility criteria for the study enrolment are outlined in Table 1. The inclusion of ALHIV, who were aware of their HIV status prior to their recruitment, was based on the endeavour to avert potential distress during data collection. Including service providers participating in this research was an advantage for gaining perspectives and interpretations of the findings from experts in that field, who may usually interact with the ALHIV on a more personal level.

**Table 1:** Eligibility criteria for research participants

Research participants	Inclusion criteria	Exclusion criteria
**Adolescents living with HIV**	(a) Aged 10 to 19 years (b) Living with HIV (c) Knowledge of their HIV status (d) Capacity to give consent (e) Consent by caregivers for participants, who are under 18 years old	(a) Unaware of HIV status (b) No consent by caregivers for participants, who are under 18 years old
**Service providers**	(a) Employed at the HIV clinic as clinical staff, HIV peer support group coordinators or counsellors for ALHIV (b) Experience of at least 1 year working with ALHIV to ensure they have sufficient competence in this topic area (c) Consent to participate in the study	(a) Students on clinical placement in the HIV clinic (b) Experience of less than 1 year working with ALHIV

The recommended sample size for phenomenological studies varies from 1 to 12 participants [62] to 5–25 participants [59]. However, Braun and Clarke [63] question the usefulness of predefined sample sizes since data saturation in reflexive thematic analysis (TA) is a subjective conception. In order to collect sufficient data for answering the research question, this study recruited 16 ALHIV and five service providers, which allowed different layers of interpretation of the drawings, including the viewpoint of the individual ALHIV, ALHIV in a group and service providers as experts working with this population. Demographic information of the participants is outlined in Tables 2 and 3.

**Table 2:** Demographic information of adolescent participants

Name*	Gender	Age**	Age** at disclosure	Disclosed by	Orphan status	School grade
Lydia	Female	13	12	Counsellor	Paternal	Standard 8
Thalandira	Female	13	11	Mother	Non-orphan	Standard 7
Shanita	Female	14	10	Mother	Paternal	Standard 6
Patuma	Female	14	12	Counsellor	Paternal	Standard 5
Chisomo	Female	15	14	Doctor	Non-orphan	Standard 5
Amina	Female	16	11	Doctor	Maternal	Standard 8
Ndaziona	Female	17	15	Mother with doctor	Paternal	Form 3
Anne	Female	18	13	Mother	Paternal	Form 2
Mishello	Male	10	8	Mother	Non-orphan	Standard 4
Mkango	Male	12	8	Mother	Paternal	Standard 6
Woimba	Male	12	10	Doctor	Non-orphan	Standard 5
Lion	Male	13	11	Doctor	Non-orphan	Standard 4
Elmore	Male	15	12	Counsellor	Paternal	Form 1
Jonathan	Male	15	12	Hospital staff	Non-orphan	Standard 6
Lovemore	Male	16	15	Doctor	Non-orphan	Form 1
Daligo	Male	18	12	Mother	Non-orphan	Dropped out at Form 1

*All names are pseudonyms chosen by the participants **Age in years

**Table 3:** Demographic information of service providers

Name*	Gender	Age**	Function at hospital	Years of experience working with ALHIV
Eveless	Female	32	Community health worker and counsellor	7
Martha	Female	60	Nurse	5
Daniel	Male	26	Nurse and mentor	8
John	Male	31	Mentor	9
Steve	Male	41	Clinical officer	10

*All names are pseudonyms **Age in years

All ALHIV were aware of their HIV status, on ART and attended an HIV peer support group. While most reported having acquired HIV through mother-to-child transmission, some were unsure of how they obtained HIV. The service providers had between 5 and 10 years experience working with ALHIV; notably two lived with HIV themselves.

### Recruitment and sampling

After obtaining ethical approval, participants were recruited from July to December 2021 with the help of ALHIV peer support group coordinators, who served as gatekeepers. Written informed consent was obtained from all participants, including from caregivers for those participants who were under 18 years old.

Purposive sampling was used for being able to collect data from participants who gained lived experiences on the same phenomenon [59]. The sample of ALHIV was drawn from those attending a hospital-based HIV peer support group. Service providers were approached directly by the researcher to identify and recruit suitable participants for the FGD.

As ALHIV have distinct characteristics that may impact their participation, they were categorised into four homogeneous groups for the FGDs (Table 4) to reduce power imbalances and to increase participant comfort and candour when discussing sensitive topics [64].

**Table 4:** Composition of the focus groups

Group	Composition	Number of participants
**1**	Female ALHIV aged 10–14 years	4
**2**	Female ALHIV aged 15–19 years	4
**3**	Male ALHIV aged 10–14 years	4
**4**	Male ALHIV aged 15–19 years	4
	**ALHIV total**	**16**
**5**	Clinical staff, HIV peer support group coordinators and counsellors	5
	**Service providers total**	**5**

### Data collection

Data were collected face-to-face between September and December 2021 using semi-structured interview guides and visual methods. A total of 32 in-depth interviews (IDIs) and five focus group discussions (FGDs) were conducted, each lasting between 50 and 165 min, resulting in 51.7 h of audio-recorded data and 25 drawings. Each of the 16 adolescents participated in two IDIs - an initial interview involving the drawing of HIV and a follow-up interview 2–4 weeks later; they also took part in four FGDs. An additional FGD was conducted with five HIV service providers (Table 5).

Employing drawing as a research method empowered the participants through active engagement in the research [65, 66] and provided visual data, which added to the depth of the interview process, as the visual products generated knowledge and concurrently served as a medium of communication [67]. This triangulation of data collection methods was chosen to gain deeper insights into the phenomena under study. Data were collected face-to-face, mostly on weekends to avoid interfering with participants’ school schedules. All interviews and FGDs took place within the HIV clinic, in a quiet room. Since the IDIs and FGDs with ALHIV were conducted in Chichewa, two trained translators, employed at the clinic but not part of the core research team, were included. Participants were informed of the translators’ roles and provided informed consent for translation and audio recording. Transcripts were subsequently produced from the recordings for analysis.

**Table 5:** Phases of data collection

Phase	Data collection method	Purpose
**Phase 1**	Initial IDI with ALHIV	- To explore the lived experiences and perceptions of ALHIV
	ALHIV’s drawing of HIV and comparison of participants’ visual products with electron microscopic images of HIV	- To investigate ALHIV’s imagination of HIV - For participants to reflect on their HIV perceptions
**Phase 2**	Follow-up IDI with ALHIV	- To review ALHIV’s experience of drawing and whether the observation of the electron microscopic images of HIV and the reflection on it conveyed changes in their perception of HIV - To link their perception with the language used for HIV - To confirm interpretations made during the initial IDI (member checking)
**Phase 3**	Four FGDs with ALHIV (divided according to age and gender)	- To discuss ALHIV’s visual products and their underlying meanings - To evaluate alternative images of HIV (electron microscopic images and HIV representations in hospital HIV books)
**Phase 4**	One FGD with service providers (Clinical staff, HIV peer support group coordinators and counsellors)	- To elucidate on and contextualise the interpretations of service providers on ALHIV’s visual products in comparison to electron microscopic images of HIV and the visual aids used during HIV counselling sessions - To suggest changes on HIV education material

### Data analysis

Reflexive TA was used for data analysis following an inductive, idiographic and iterative approach, including the identification and grouping of themes at a latent level of interpretation to arrive at concluding themes, which were then translated into a narrative account [68]. The drawings, conveying meaning in non-verbal visual voices, were contextualised by the participants’ narratives, which added meaning through their own reflections and interpretations [69]. This helped to reduce interpretation bias, since the interpretations of the visual products remained close to the narrative meaning participants attached to them [70].

### Ethical considerations

The research followed the ethical principles of the Declaration of Helsinki [71]. After receiving permission to conduct the study from the research setting, ethical approval was granted both at the faculty level by the Faculty of Health and Medicine Research Ethics Committee at Lancaster University (UK) and at the national level by the National Committee on Research in the Social Sciences and Humanities under the National Commission for Science and Technology in Malawi. Since ALHIV were the main participants in this study, the process of informed consent adhered diligently to the ethical principles of autonomy, non-maleficence, beneficence and justice [72]. To ensure that participation was entirely voluntary and not coercive, prospective participants received adequate information on the research process, the benefits and possible risks or anticipated discomforts, the support provided in case of distress and the right to withdraw their consent without any negative consequences [73]. Furthermore, the information for ALHIV and their caregivers was conveyed in simple terms, using their local language to assure comprehension. To avoid ethical challenges regarding confidentiality and privacy, the participants did not use their names on their drawings [74]. Because the topic of HIV is highly associated with stigma, anonymity and confidentiality were crucial and applied to recorded, written and visual data. With the use of pseudonyms, the participants’ privacy and identity were always protected.

### Trustworthiness and rigour of the research

This study employed Lincoln and Guba’s assessment criteria for research trustworthiness and rigour, including credibility, transferability, dependability and confirmability [75]. Considering the emic stance of the researcher and the subjective nature of hermeneutic phenomenological studies through their interpretive character, the researcher’s continuous reflexivity to acknowledge the direct influences on the study has been recorded in a reflective journal [76].

## Results

After data analysis, three main themes were identified (characteristics of HIV, language used for HIV and understanding HIV), which elucidate how ALHIV describe, perceive and imagine HIV in their bodies, as well as how these interpretations influence their emotional wellbeing. Also, the views of service providers on these themes are illuminated.

### Characteristics of HIV

ALHIV described HIV as an organism with gender identity and nutritional needs to function:

*“[HIV eats] the same as we eat. When we are taking medication*,* the virus is very weak*,* so it can’t eat.”* (Lydia, female, 13).

**Fig. 1:**
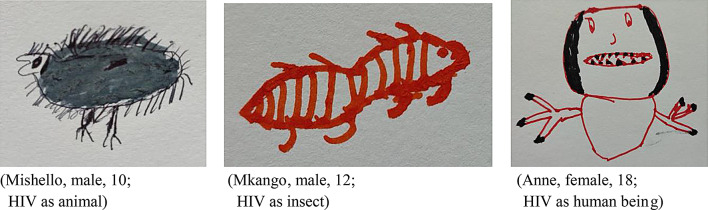
HIV perceptions

While the majority of participants drew HIV as an animal or insect, some portrayed it in form of a human being, existing and “fighting” within their bodies (Fig. 1):

*“Because it’s HIV*,*…I always think how HIV enters my body.…Because the virus wants to affect my body.”* (Lion, male, 13).

Furthermore, HIV was drawn with features, such as a face with eyes, nose, ears, mouth and teeth, a body, feet and arms. Hence, HIV has been anticipated existing with functional senses, including sight, taste, smell and hearing. Intriguingly, for some participants HIV was able to communicate with other HIVs or CD4 cells in their body:

*“Because there are many*,* not a single virus. They may have some disagreements…when they are eating something*,* they may quarrel.”* (Mkango, male, 12).

Those perceptions were related to their understanding of HIV requiring to adapt to, function and move in a habitat, which in this case is the human body. Regarding ALHIV’s imagination of the size of HIV, most understood the concept of an invisible pathogen within their body, and as such, for them HIV was invisible. However, some participants thought that HIV might be visible, having a size from 1 to 14 cm. Moreover, most participants imagined HIV in red colour due to its habitation in blood, others believed it would be black, transparent, grey or white. Those imaginations of HIV left emotional footprints in ALHIV:

*“I am feeling sad*,* because the virus will always remain in the body and making me busy taking the ART.”* (Amina, female, 16).

During the FGDs, participants inspected all drawings produced during the research and recognised ALHIV’s different imaginations and perceptions regarding HIV, which they attributed to HIV’s invisible nature. However, the service providers ascribed the explanations given during hospital counselling sessions and the local term for HIV “Kachilombo” to participants’ drawings and perceptions. Nevertheless, these HIV drawings reflect on ways ALHIV try to make sense of HIV and what HIV could do to their bodies, yet, differences between the two age groups (10–14 years and 15–18 years) - or between female and male participants - were not identified. Several participants indicated the important role that the term “Kachilombo” played in influencing their HIV imagination.

### The Language used for HIV

The language used for HIV includes the language of silence and secrecy, the local term used for HIV “Kachilombo” and war metaphors.

Participants vividly described their reality concerning HIV-related stigma, with their constant fear of segregation and rejection, in case others may get to know about their HIV status. Though most participants got to know their HIV status during counselling sessions by healthcare providers, their caregivers strongly advised them to conceal their HIV status to evade discrimination:

*“My Mom told me that I should not tell anyone [about my HIV status]*,* it’s confidential. Only my Mom and I know.”* (Mkango, male, 12).

Consequently, their autonomy for telling others about their HIV status was taken and they felt a need for secrecy and hiding their ART, so that even close family members were often not aware of their HIV status. This silence around HIV restricted them from questioning further. Others expected harsh punishments if they decided to openly discuss their HIV status:

*“They would beat me. My parents told me to go and play and not to talk about my HIV status.”* (Mishello, male, 10).

Also, in view of disclosing their HIV status to intimate relationships, the older ALHIV mentioned the need for secrecy as they feared rejection and discredit due to their HIV-positive identity. However, frequently the support group was mentioned as an important support structure, providing a safe environment for meeting peers, asking questions and discussing HIV-related challenges, making living in secrecy there redundant.

**Fig. 2:**
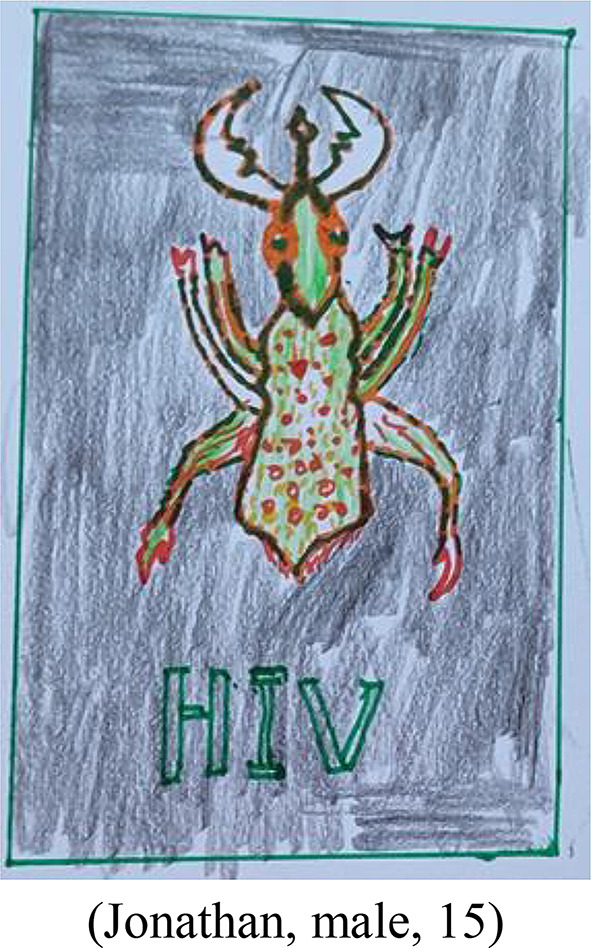
HIV in form of a scorpion

Since the term “Kachilombo” is also used when talking about insects or small animals in Malawi, the participants repeatedly depicted HIV as such and thus, they explained the different meanings and attributes of “Kachilombo”:

*“Kachilombo means different things…Something which is dangerous that can bite people. Kachilombo can affect people or can make people sick.”* (Amina, female, 16),

As a result of HIV being a highly sensitive and stigma-related taboo topic, the understanding of “Kachilombo” as euphemism for HIV clearly shaped participants’ perceptions and imaginations of HIV, which was also mirrored in a drawing of HIV as a scorpion (Fig. 2).

This shows how language translates into participants’ understanding and imagination of HIV as insect or small animal. The participants further explained that they conceive “Kachilombo” as a dangerous, frightening and invisible invader for which no cure was available. Attributable to its double meaning, the term “Kachilombo” was also perceived as reinforcing discrimination:

*“Better using HIV than Kachilombo. For our culture here in Malawi*,*…Kachilombo can give people different ideas and maybe psychological problems as it is something scary and that can destroy life.”* (Anne, female, 18).

The service providers added that understanding “Kachilombo” as a single HIV, linked to participants’ drawings of a single virus, was considered inaccurate information, especially when communicating the concept of HIV multiplication and viral load:

*“Kachilombo as a single virus is confusing because they don’t think that HIV does multiply.”* (Daniel, male, 26).

For making sense of scientific ideas, participants frequently applied war metaphors, such as “fighting” and “attacking” of HIV and CD4 cells, to explain the relationships and battles between them. When elaborating on the positive effects of ART on the CD4 cells for winning the battle against HIV, ALHIV commonly used the “fighting” terminology:

*“So*,* if you are taking medication*,* the CD4 will have power to fight the virus and it [HIV] will get weak and sleep. So*,* you cannot get sick if you adhere to medication.”* (Lydia, female, 13).

In addition, the CD4 cells were labelled as HIV’s “enemy” and the comprehension of “HIV as a killer” was presented as follows:

*“[HIV has a mouth] to kill the white blood cells in the body.”* (Lovemore, male, 16),

The use of war metaphors created an impression of ALHIV living with a constant battle in their bodies. The utilisation of war metaphors has been adopted from HIV lessons received at school, but also during hospital counselling sessions, where fear-based messages are used to impart knowledge and reinforce ART adherence. This has been confirmed in the FGD with the service providers, who explained ALHIV’s linguistic presentation of “HIV as a killer” as a metaphorical product of what they learnt about HIV, especially at school, where outdated information would be used for teaching, ignoring the fact that ART enables people to live a “normal” life with HIV:

*“…the teacher told us that ‘If you have HIV*,* you are dead! HIV is a killer!’.”* (Steve, male, 41).

Service providers further understood the negative impact of threatening messages, however, they also believed that downplaying HIV as harmless might cause non-compliance to therapy. With the availability of ART, which allows people to live with HIV as a chronic condition, the importance of what and how people learn about HIV, needs to be considered, as it affects ALHIV’s perception about HIV in their body, but also their sense of an “HIV-positive” identity.

### Understanding HIV

Evidence from ALHIV’s stories suggest that the initial knowledge about HIV has been mainly acquired at schools, whereby they also learnt from community interactions and in hospitals.

Some participants evaluated the content of HIV lessons inadequate due to schools’ main focus on HIV prevention and presentation as “killer”, closely linked to inevitable death:

*“…because teachers don’t have basic knowledge about HIV. They have this information of HIV*,* which is wrong…the way the teacher was presenting the HIV topic in class caused more stress to me.”* (Anne, female, 18).

While in schools the educational focus is mainly set on HIV prevention, leaving pupils living with and being affected by HIV behind, the emphasis of HIV education in hospitals is placed on “living positively with HIV”, which may contribute to tensions between ALHIV and HIV-negative adolescents.

According to the participants’ experiences, due to inaccurate HIV information, people in communities still construct HIV as a dangerous “killer disease”, linked to a premature death sentence, instigating the need to sideline the affected population. ALHIV were frequently subjected to people negatively debating topics around HIV, guided by misinformation, such as HIV transmission through “normal” contact, HIV changing the physical appearance of PLHIV or that women living with HIV would always deliver sick babies. These beliefs fuelled the fear of HIV and PLHIV, and consequently led to stigma and discrimination. Nevertheless, some ALHIV assumed that accurate information about HIV could reduce the level of discrimination.

In contrast to the HIV education attained at school, ALHIV evaluated the knowledge gained in hospitals as more effective due to the inclusion of information regarding “living positively with HIV”, which distinction made them feel less threatened by HIV and more equal to HIV-negative peers. Still, despite praising ART’s effectiveness for their own benefit, participants were unaware of the “Undetectable viral load equals untransmittable HIV infection” (“U = U”) or “ART as prevention” concepts, increasing ART’s advantage to intimate partners and their future children. Some participants reported uncertainty about these inconsistencies in HIV education, particularly regarding their life expectancy. This confusion was also noted concerning ALHIV’s beliefs about HIV`s duration in their body with most participants understanding HIV as a chronic, lifelong condition, and others thinking that HIV would disappear one day.

**Fig. 3:**
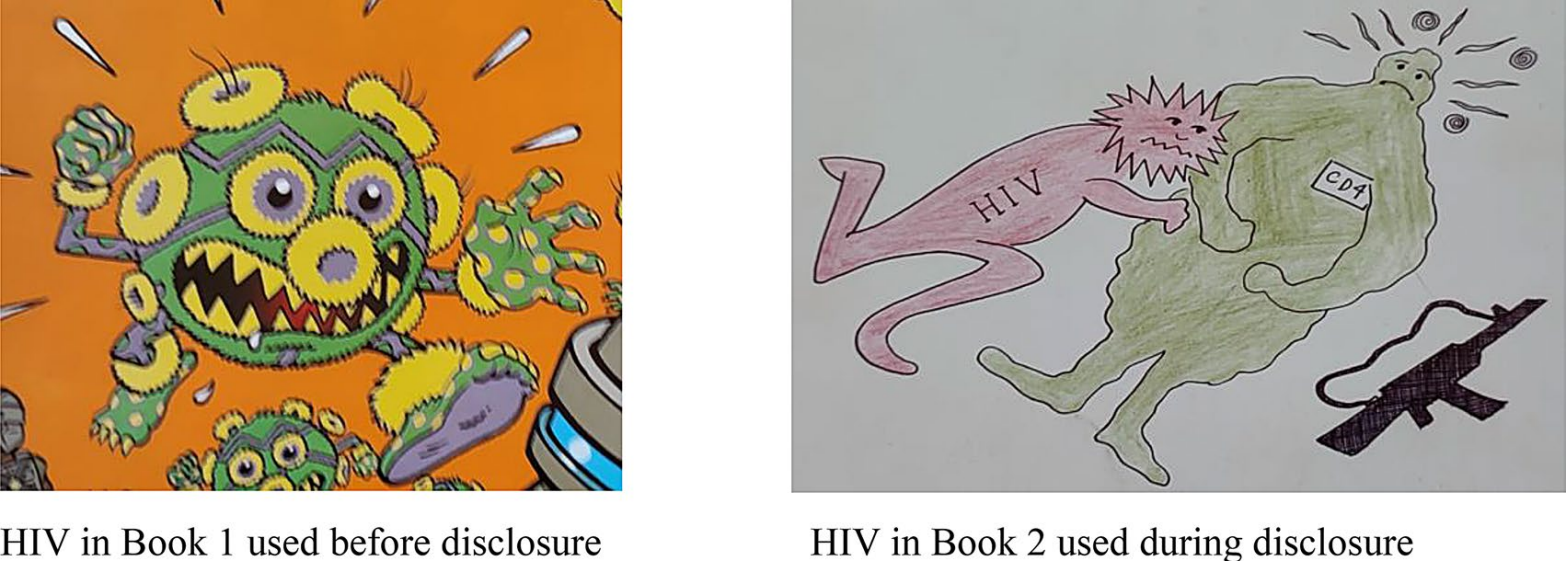
HIV drawings in books

**Fig. 4:**
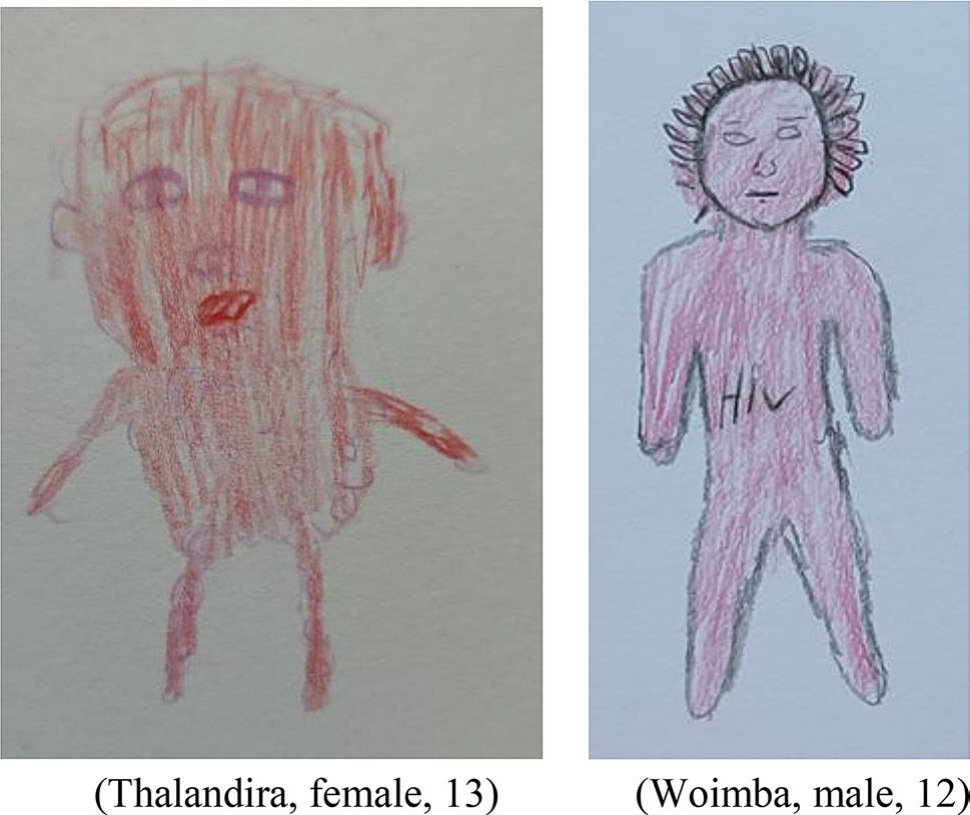
HIV perceptions like HIV in Book 2

Evidently, by placing emphasis on the aspect of living positively with HIV, the information provided at hospitals reduced ALHIV’s anxiety of HIV, ameliorating the information received from communities and schools. Therefore, service providers proposed aligning the HIV education offered at schools and hospitals to address this bottleneck, resulting in a population with an equal knowledge base. However, because participants mentioned the influence of hospital HIV books on their imagination of HIV, those books were, together with the participants, assessed. The following two images of HIV were taken from educational material ALHIV had access to (Fig. 3):

Most participants evaluated the HIV representation in both books as dangerous, compromising their emotional wellbeing. Interestingly, few participants perceived the scary HIV images as beneficial to enforce ART adherence. Nevertheless, the use of pictorial education material within these books clearly inspired some participants in view of their choice of drawings. As such, two drawings were produced almost identical to the image of HIV in Book 2 (Fig. 4).

Most participants preferred less dangerous, kinder HIV images in those books to alleviate fears and avoid distress and depression. Furthermore, participants elaborated on the frightening images of HIV presented in teaching material, causing avoidance behaviour by HIV-negative people:

*“That can make people think that those who have HIV and AIDS*,* they are people that cannot be friendly. That can promote stigma and discrimination.”* (Anne, female, 18).

Importantly, the service providers also pointed out that a positive perception and imagination of HIV may enhance ALHIV’s acceptance of themselves with this condition, mitigating fear and depression, which in turn, would improve their health.

**Fig. 5:**
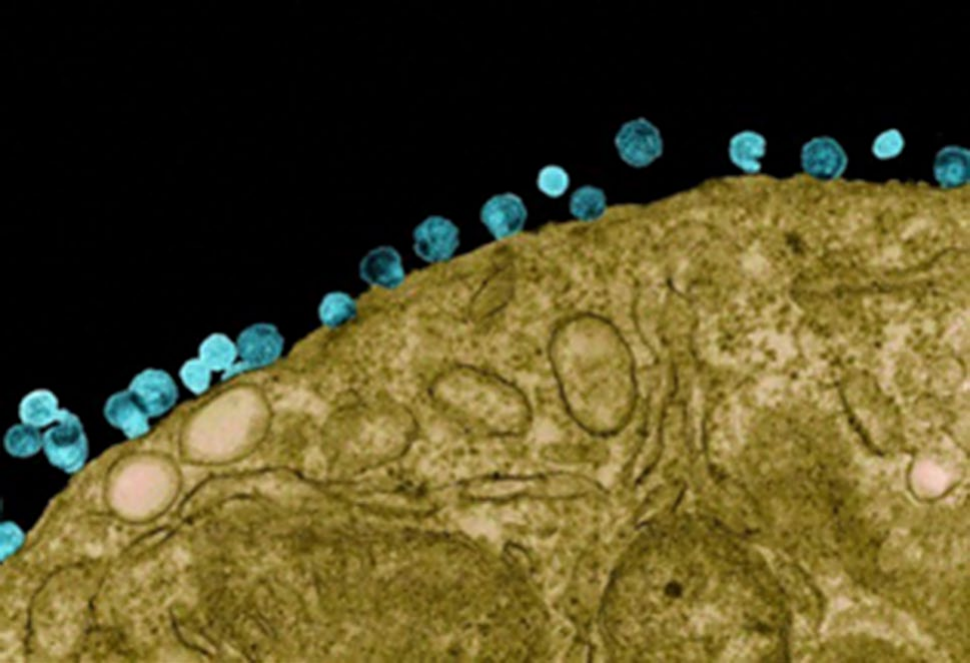
HIV under the electron microscope [77]

However, a transformation occurred during the research, when the following image of HIV under an electron microscope was exhibited (Fig. 5):

For all participants it was their first-time viewing HIV in this form, and they appreciated gaining this insight while recognising differences between HIV on their drawings, in the hospital books and the photo:

*“I am feeling much better*,* because I know now how the real virus looks like.”* (Thalandira, female, 13).

Participants perceived the scientific HIV image as friendlier, attributed to the absence of body parts:

*“In the past I thought that the virus looks dangerous and when I saw the real picture*,* I saw that the virus has no eyes*,* no legs*,* it is the same like cells in the body. I feel now better.”* (Anne, female, 18).

Some participants even advised including the scientific HIV image in hospital and school books in order to eliminate misconceptions.

ALHIV’s emotions were induced by their HIV perceptions and formed by formal and informal HIV information. Presumably attributed to the secrecy and silence around HIV participants did not talk directly about their emotions. Furthermore, sadness, anger and uncertainty were stated as negative emotions with fear most frequently mentioned in the context of forgetting ART, of illness and death, of discrimination, when they got disclosed and of letting others know their HIV status. Yet, ALHIV did not directly reveal the fear of HIV itself in their bodies, despite the often-scary creatures brought onto paper. Nevertheless, positive emotions, such as happiness were reported in view of support and the hope for a successful life.

## Discussion

This study was able to identify themes central to how ALHIV imagine HIV and in what ways these images and the language used for HIV impact on their emotional wellbeing, perceptions and lived experiences. The participants’ narratives were augmented by their insightful drawings of HIV and explanations thereof.

Most ALHIV imagined HIV as a living creature with human, animal or insect features, a gender identity, functional senses and nutritional needs. The meaning they attached to HIV’s personification was that of a dangerous and harmful creature in their bodies, especially when ART was not taken, because then HIV was believed to be awake, moving and performing a fight within their bodies. The personification of invisible pathogens invading a human body illuminated their existence as physical and at the same time as a psychological threat [78]. Similarly, patients in India [79], Canada [80] and Australia [81] confirmed comparable perceptions of different types of cancer as intruders, taking over their identity.

Illness personifications may be influenced by culture, gender and age, however, also Nicaraguan PLHIV referred to a “sleeping HIV” through ART as a controlling agent [82]. Moreover, in a study exploring illness meanings among female PLHIV aged 18–24 in the US, HIV has been portrayed as a “gremlin”, implying the perceived “physical, social, and emotional threats posed by HIV” [47].

Comparable perceptions of disease were observed in drawings produced by Dutch adults, who depicted their tumours in the form of monsters [46] and in drawings of pain among 5–18 year old Canadians [83]. These findings call into question the sources of those perceptions and in how far the negative, threatening personifications of ill-health serve the purpose of imparting knowledge ethically, considering the implications for individuals’ emotional wellbeing. In order to dissociate from the view of cancer as an “external invader”, Lucas [84] suggested placing the emphasis on strategies to reduce the multiplication of cancerous cells. With regard to HIV, a similar approach could be applied in view of ART for controlling the multiplication of HIV (“U = U”).

Regarding the impact of these perceptions on participants’ emotional wellbeing, most participants perceived a friendly imagination of HIV linked to better emotional wellbeing and facilitating good ART adherence. Therefore, they proposed the inclusion of friendly pictorial HIV representations in teaching material. While this could ameliorate the misinformation received, extending these findings to school curricula could close the knowledge gap between ALHIV and their HIV-negative peers and thus, provide an equal knowledge base, with the potential of reducing HIV-related stigma and discrimination. Considering emotions as catalysts for health behaviour, pictorial HIV representations need to be selected cautiously. Other participants, in contrast, stressed the importance of imagining HIV as a frightening creature, which might positively impact ART adherence due to fear, instigating pressure to adhere to ART and thus, find peace in forcing HIV into a nonviolent state. With this in mind, it might be argued that a one size fits all educational approach may not work for all ALHIV. Therefore, in order to improve ALHIV’s understanding of HIV, accurate and uniform knowledge about HIV may reduce their misperceptions and enhance their emotional wellbeing.

Their descriptions, perceptions and representations of HIV were a result of the language used for HIV, the exposure to conflicting HIV information and pictorial HIV representations in hospital HIV books, which further influenced ALHIV’s emotional wellbeing. Language, as an important tool for health communication [85], has an impact on individuals’ perceptions [86] and emotions [87]. Terminologies used for certain conditions evidently impact on health-seeking behaviour and treatment adherence, but also on mental and physical health outcomes [50]. Attitudes towards HIV are shaped by the terms and images used for HIV, which generate distinctive, abstract realities, and reflect upon individuals’ understanding and emotional response to it [88, 89].

Regarding the language used for HIV, due to stigma and discrimination, the silence and secrecy around HIV imparted shame and fear of openly requesting HIV information, and even worse, rendered ALHIV unwilling to disclose their HIV status, which contributes to the continuation of HIV onward transmission. Also, caregivers of 11–13 year old ALHIV in Uganda and Zimbabwe counteracted HIV-related stigma and discrimination by the reinforcement of reticence, creating a form of double life and subduing the children’s autonomy [90]. Employing silence and secrecy to control stigma and discrimination caused a perception of being different and frequently promoted poor ART adherence. With growing maturity ALHIV need to learn HIV disclosure strategies to meet responsibilities while mediating potential consequences [91]. Since the root causes of the dilemma lay in HIV-related stigma and discrimination, efforts directed solely at the ALHIV population may not tackle these challenges and therefore require a wider approach, including caregivers, schools and communities.

Furthermore, the figurative and metaphorical term used for HIV in Malawi, “Kachilombo”, that can be translated as “small beast” [92], “small animal” [93], “insect” [94] or “virus” [95], provoked confusion due to its multiple meanings and created misperceptions regarding HIV as personification and anthropomorphisation in ALHIVs’ bodies. This shows how African vernaculars convert medical terminologies for an invisible pathogen to symbolic, figurative language [95]. Because communication about sex and HIV remains taboo in SSA, restricting related verbal expressions, the application of indirect terminologies aids in breaking the culture of silence surrounding these topics [96]. Nevertheless, the utilisation of these terminologies has been contested as they may contribute to misperceptions and stigma, and compromise HIV status disclosure [97], as well as HIV testing and treatment [98].

Just as language contributes to knowledge conception, it likewise serves emotive functions, which build different perceptions [99]. This was confirmed by a study examining the terminologies used for HIV in the Democratic Republic of the Congo, where HIV labels, such as “insect” or “animal” created stigmatising behaviours through the differentiation of people living with or without the “insect” or “animal” in their bodies [100].

Since terminologies play a crucial role when communicating HIV-related topics, the use of culturally appropriate and concurrently technically correct information is essential, especially in regions with high HIV prevalence [101]. With this understanding, the exclusion of local HIV vocabularies in the UNAIDS guidelines on HIV terminologies [102] disregards their importance in guiding HIV knowledge and behaviour.

The utilisation of war metaphors when communicating information about HIV further amplified ALHIV’s misconceptions. Despite not visually represented on participants’ drawings, HIV was verbally designated as the major actor of constant war scenes within their bodies. The participants frequently utilised war metaphors describing their sense-making of HIV with its physical effects and with ART as survival strategy. This language of war has been induced by teachers during HIV lessons, where the emphasis of “HIV as a killer” leads to erroneous assumptions of living with HIV in the era of ART, enabling people to live a “normal” life with HIV. Similarly, the chosen language employed in hospital counselling sessions included fear-based messages to impart knowledge and reinforce expected behaviours. These elaborations, together with the depiction of additional “HIV monsters” in hospital HIV books, contributed to a representation of HIV as a destructive and menacing entity.

Although interpreting diseases from a war perspective is considered controversial in literature [92], the advantage of employing war metaphors was found to increase patients’ and healthcare workers’ optimism regarding the process of recovery and to improve resource mobilisation for research [103]. Yet, while the focus of war terminologies mainly lies on the physical aspects of illness, it neglects the psychosocial and spiritual attributes of ill-health, making their use ethically disputable [104].

The emotional consequences through the use of war terminologies include negative feelings, such as fear, guilt, shame and demotivation [105]. In line with this, the UNAIDS guidelines on HIV terminologies [102] discourage its use for HIV communication to avoid the inference from a “war against HIV” to a “war against PLHIV”. For evading harm, alternatively to the war terminologies currently used, a simple, honest and culturally appropriate language may promote mutuality, equality and the same time the comprehension of HIV as presently chronic, manageable condition [106].

In addition to verbal HIV education, pictorial materials were used in the hospital setting to help explain HIV. Pictorial illness representations of pathogens are useful for explaining complicated health information, especially in populations with reduced literacy skills [107]. An integrative review, including adults, found that visual education material notably enhanced health literacy [108]. However, for effective visual communication, decisions on the choices of pictorial images need to consider its understandability and interpretation, as well as the ethical and emotional appropriateness [109]. The utilisation of scary HIV representations, such as monsters to explain HIV, may create misconceptions [37, 38].

According to Manches and Ainsworth [109], children have the cognitive capacity to understand the biology of invisible viruses, provided their representation is pertinent. Exposing the participants to scientifically produced HIV images illuminated their perceptions in several ways. All adolescent participants valued the exposure to these images and most revealed positive emotions towards HIV’s simple and friendly appearance on these pictures. Notably, they perceived scientific HIV images less distressing, and therefore suggestions of integrating those HIV images into educational material were made. Comparable remarks were made by Malawian ALHIV, who participated in an art therapy workshop in 2016 [45]. In spite of the advantages of blending verbal and visual properties into HIV education, these sources require quality control and pretesting in view of their effectiveness regarding the context and the target groups.

Considering that ALHIV’s perceptions of HIV and their emotional wellbeing were strongly impacted by the language used for HIV, the conflicting HIV information and the effects of pictorial HIV representations, HIV communication and education strategies may need to be reviewed and evaluated to promote emotional wellbeing and thus, healthier behaviour, including ART adherence. Since education can transform HIV perceptions and facilitate social change, a more holistic and sensitive educational approach - employing positive reinforcement strategies - might be useful not only for populations living with HIV, but equally for those responsible for HIV-related stigma and discrimination.

### Limitations

The methodological strengths of this research included the use of drawings, which facilitated ALHIV’s nonverbal expression and enriched discussions about their perceptions of HIV. The additional perspectives from service providers further validated and deepened the understanding of the phenomena. Nonetheless, the study has several limitations. While qualitative research aims for transferability rather than generalizability, the transferability of these findings may be limited because participants were ALHIV enrolled in HIV peer support groups who receive education and psychosocial support. Their experiences might differ from those ALHIV without such access. Future studies could include a more diverse sample of ALHIV outside peer support contexts to enhance transferability. Additionally, as participants reportedly acquired HIV perinatally, the findings may not fully represent adolescents who acquired HIV through other routes; subsequent research could explore these different subgroups. Finally, involving interpreters may have led to some loss of nuance in euphemisms or culture-specific expressions during translation. To mitigate this, future research might incorporate bilingual researchers or involve back-translation procedures to preserve meaning more accurately.

### Recommendations

Regarding the HIV education in hospitals, a more holistic approach, considering the appropriateness of pictorial elements and the language used may enhance ALHIV’s understanding and perceptions of HIV and thus, positively impact on their emotional wellbeing and adherence to ART. Therefore, it is important to involve ALHIV in the evaluation of current learning material, and equally during the planning and development of educational sources [110]. HIV education is the basis of HIV prevention and control, but to stop the spread of HIV, the spread of HIV-related stigma and discrimination needs to end. In light of this, intensified efforts need to promote contemporary HIV knowledge and challenge misconceptions, as well as negative stereotypes and behaviours regarding HIV since these may undermine efforts to reduce HIV onward transmission. This research exhibited that drawing was a useful tool for collecting data, however, integrating drawing as an interactive participatory learning approach could be beneficial to enhance the HIV learning experience and substitute HIV perceptions [46].

Future research could further explore HIV perceptions among different populations, such as adult PLHIV, adolescents not living with HIV, ALHIV not accessing support services, as well as ALHIV who acquired HIV behaviourally. With specific consideration of the respective terminologies used for HIV, future studies could enhance the understanding of the preferences and learning needs in different populations to tailor appropriate HIV education material. Also, the HIV perceptions of caregivers, healthcare workers and teachers could provide a greater variety of perspectives in order to steer a multidisciplinary approach when aiming for novel and appropriate care strategies.

## Conclusion

This phenomenological study provides deeper insight into the perceptions of HIV among ALHIV in Malawi. These perceptions appear to be shaped by HIV education and the language used to describe HIV, including the local terminology, war metaphors, and the broader silence and stigma surrounding the condition. The findings illustrate tensions between formal HIV education, local understandings, and their influence on the emotional wellbeing of ALHIV. Based on these insights, the study suggests the value of reviewing and contextualising HIV educational materials used in schools, communities, and healthcare settings, with the aim of improving health literacy, supporting emotional wellbeing, and addressing HIV-related stigma and discrimination.

## Data Availability

The data sets are stored with Lancaster University, but relevant data is also provided within the manuscript.

## Abbreviations

ALHIV: Adolescents living with HIV
ART: Antiretroviral Therapy
FGD: Focus Group Discussion
HIV: Human Immunodeficiency Virus
IDI: In-depth Interview
PLHIV: People living with HIV
TA: Thematic Analysis
“U = U”: “Undetectable viral load equals untransmittable HIV infection”
WHO: World Health Organization
